# Associations between the Neutrophil-to-Lymphocyte Ratio and Diabetic Complications in Adults with Diabetes: A Cross-Sectional Study

**DOI:** 10.1155/2020/6219545

**Published:** 2020-04-28

**Authors:** Heng Wan, Yuying Wang, Sijie Fang, Yi Chen, Wen Zhang, Fangzhen Xia, Ningjian Wang, Yingli Lu

**Affiliations:** ^1^Institute and Department of Endocrinology and Metabolism, Shanghai Ninth People's Hospital, Shanghai Jiao Tong University School of Medicine, Shanghai, China; ^2^Department of Ophthalmology, Shanghai Ninth People's Hospital, Shanghai Jiao Tong University School of Medicine, Shanghai, China

## Abstract

**Objective:**

The neutrophil-to-lymphocyte ratio (NLR) is an inexpensive and easily measurable laboratory index indicating systemic inflammation, while the application of many other inflammatory markers has been limited in daily clinical practice. However, large population studies about investigating the associations of the NLR level with diabetic complications including cardiovascular and cerebrovascular diseases (CVD), diabetic kidney disease (DKD), and diabetic retinopathy (DR) in the same population were limited. The aim of our study is to evaluate the associations between the NLR level and the prevalence of CVD, DKD, and DR in adults with diabetes simultaneously.

**Methods:**

A cross-sectional survey of 4,813 diabetic adults was conducted in seven communities in China. Persons underwent several medical examinations, including the measurement of anthropometric factors, blood pressure, routinely analyzed leukocyte characteristics, glucose, lipid profiles, urine albumin/creatinine ratio, and fundus photographs.

**Results:**

Compared with the first quartile of the NLR level, the odds of having CVD was significantly increased by 21% for participants in the highest quartile (OR 1.21; 95% CI 1.00, 1.47) (*P* for trend < 0.05). Similarly, the prevalence of DKD among participants in the highest quartile of the NLR level was significantly increased by 150% (OR 2.50; 95% CI 1.95, 3.19) (*P* for trend < 0.05). However, no association was found between the NLR level and the prevalence of DR (*P* for trend > 0.05). These associations were all fully adjusted.

**Conclusions:**

A higher NLR level was associated with an increased prevalence of CVD and DKD, other than DR, in diabetic adults.

## 1. Introduction

Type 2 diabetes mellitus (T2DM) has become a serious threat to global human health because of its vascular complications which are associated with increased disability, frailty, and reduced life expectancy, including cardiovascular and cerebrovascular diseases (CVD), diabetic kidney disease (DKD), and diabetic retinopathy (DR) [1–3]. A recent review reports that half of diabetic adults present with diabetic microvascular complications and 27% with macrovascular complications [4]. Thus, considerable attention has been directed towards early diagnosis and effective surveillance of diabetic complications.

Chronic inflammation has been considered the potential pathogenesis responsible for the development of diabetic complications [5–7]. However, the application of many inflammatory markers has been limited in daily clinical practice because of their costs and technical difficulties in measuring. It is noteworthy that the neutrophil-to-lymphocyte ratio (NLR), an easily measurable and inexpensive laboratory index calculated by routinely analyzed leukocyte characteristics, combines the negative effects of neutrophils on endothelial damage with the antiatherosclerotic role of lymphocytes [8]. Therefore, the NLR has been considered a convenient indicator for systemic inflammation [9, 10].

Studies have shown that low NLR may be considered a novel surrogate marker of DN in early stages and a predictor of lower risk for hospitalizations in hemodialysis patients with diabetes [11, 12]. Some other studies have implicated NLR as a risk factor of diabetes and its complications [13, 14]. However, the studies with large sample participants were still limited and conclusions were conflicting. For example, Ciray et al. suggested that NLR was not associated with the pathogenesis of DR [15] and Gijsberts et al. considered that NLR was of a limited predictive value in prediction of cardiovascular mortality [16]. In addition, to the best of our knowledge, no studies have evaluated the associations of NLR with macro- and microvascular complications in the same diabetic population. Thus, in this large community-based sample study, we aimed at investigating the associations between the NLR level and the prevalence of CVD, DKD, and DR simultaneously in adults with diabetes.

## 2. Materials and Methods

### 2.1. Study Design and Participants

**Figure 1:**
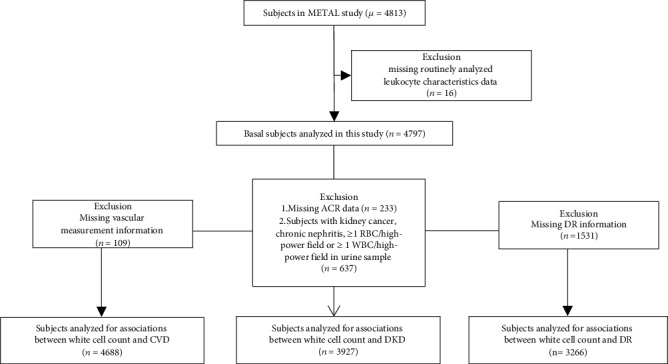
Flowchart of sampling frame and participants selected from the METAL study.

Participants who have been previously diagnosed with T2DM according to the diagnostic criteria for diabetes proposed by the Chinese Diabetes Society [17] and registered in the platform in the community healthcare center were enrolled in METAL study (Environmental Pollutant Exposure and Metabolic Diseases in Shanghai, Trial registration: ChiCTR1800017573, http://www.chictr.org.cn. Registered 04 August 2018) from seven communities in Pudong and Huangpu District, Shanghai, China. The age of the citizens was ≥18 years old and had lived in the current area for ≥6 months. 4,813 subjects with diabetes received examination in 2018. Participants who missed routinely analyzed leukocyte characteristics results were excluded (*n* = 16). We then excluded the participants missing vascular measurement information (*n* = 109); missing ACR data (*n* = 233) or subjects with kidney cancer, chronic nephritis, ≥1 RBC/high-power field, or ≥1 WBC/high-power field in urine sample (*n* = 637); and missing DR information (*n* = 1531), respectively. Finally, the number of participants who were involved in the analyses for associations of the NLR level with CVD, DKD, and DR was, respectively, 4688, 3927, and 3266 (Figure 1).

The study protocol conformed to the ethical guidelines of the 1975 Declaration of Helsinki as reflected in a priori approval by the Ethics Committee of Shanghai Ninth People's Hospital, Shanghai Jiao Tong University School of Medicine. Written consent was obtained from all the participants in our study.

### 2.2. Measurements

The questionnaire about demographics, medical history, family history, and lifestyle factors was filled out by the same trained personnel in the SPECT-China study [18, 19] during the interview. Clinical examinations, including height, weight, and blood pressure, were measured according to a standard protocol as before [20, 21]. Body mass index (BMI) was calculated as the weight in kilograms divided by the height in meters squared. We defined current smoking as having smoked at least 100 cigarettes in the lifetime and smoking cigarettes currently [22].

Overnight fasting blood (at least 8 h of fasting) was obtained between 6:00 and 9:00 am, which was refrigerated immediately and sent to a central laboratory for measuring in two hours. Routinely analyzed leukocyte characteristics including leukocyte, neutrophil, lymphocyte, and monocyte level were measured with SYSMEX XS-800i. NLR and MLR levels were calculated. Fasting plasma glucose (FPG), serum creatinine, total cholesterol, triglycerides and high- (HDL) and low-density lipoprotein (LDL) were detected with Beckman Coulter AU 680 (Brea, USA). Glycated hemoglobin (HbA1c) was tested using high-performance liquid chromatography with MQ-2000PT, (Shanghai, China). Morning urine samples were collected in the refrigerator immediately to measure the levels of urine albumin and creatinine with Beckman Coulter AU 680 (Brea, USA); then, the urine albumin to creatinine ratio (ACR) was calculated. Common carotid artery (CCA) plaque was assessed by a Mindray M7 ultrasound system (MINDRAY, Shenzhen, China) with a 10 MHz probe. Participants were diagnosed with DR by remote reading form ophthalmologists, using retinal fundus photography, Topcon TRC-NW400 Non-Mydriatic Retinal Camera (Oakland, USA) as before [21, 23].

### 2.3. Outcome Definition

The definition of dyslipidemia was that total cholesterol ≥ 6.22 mmol/L (240 mg/dL), triglycerides ≥2.26 mmol/L (200 mg/dL), LDL ≥ 4.14 mmol/L (160 mg/dL), and HDL < 1.04 mmol/L (40 mg/dL) or self-reported previous diagnosis of hyperlipidemia, according to the modified National Cholesterol Education Program-Adult Treatment Panel III [24].

The outcome CVD was defined as previously diagnosed with coronary heart disease, stroke, or peripheral arterial disease, which were recorded in the registration platform. The definition of present CCA plaque was subjectively causing a relative diameter narrowing ≥25% according to the Framingham Heart Study [25].

The estimated glomerular filtration rate (eGFR) was calculated by the Chronic Kidney Disease Epidemiology Collaboration equation for “Asian origin”. The definition of DKD was that ACR ≥ 30 mg/g and/or eGFR < 60 mL/min per 1.73 m^2^, suggested by the American Diabetes Association statement [26].

The DR classification was DR stage 0 (no abnormalities), DR stages 1 to 3 (nonproliferative DR, intraretinal microaneurysms, hemorrhages, venous beading, and prominent microvascular abnormalities), and DR stage 4 (proliferative DR, neovascularization or vitreous/preretinal hemorrhages) in accordance with the “Global Diabetic Retinopathy Project Group” [27].

### 2.4. Statistical Analysis

IBM SPSS Statistics, Version 22 (IBM Corporation, Armonk, NY, USA), was used in the current analysis. *P* value (two sided) <0.05 indicated significance. Continuous variables were expressed as the mean ± SD, and categorical variables as percentages (%) or median (interquartile range). The NLR level was divided into quartiles. Logistic or linear regression analysis was performed for measurement of the trend of variable changes across the NLR level quartiles, providing unadjusted *P* values.

A regression test was used to detect the associations between the NLR level quartiles and diabetic complications. Data were summarized as odds ratios or regression coefficients (95% CI). In the analyses, the levels of urinary ACR were logarithmically transformed to achieve a normal distribution. The associations of the NLR level quartiles with the prevalence of CCA plaque, CVD, DKD, and DR were tested by binary logistic regression analyses. Linear regression analysis was used to test the associations of the NLR level quartiles with Ln ACR and eGFR. The associations of the NLR level quartiles with NPDR and PDR were analyzed by multinomial logistic regression. Receiver operating characteristic (ROC) curve analysis was used to compare the prognostic powers of the neutrophil, lymphocyte, and the NLR level for DKD.

Sensitivity analyses were performed. We evaluated the associations between the leukocyte, neutrophil, and lymphocyte level quartiles and the prevalence of CCA plaque and CVD in Supplementary Table 1, the associations between the leukocyte, neutrophil, and lymphocyte level quartiles and the prevalence of DKD in Supplementary Table 2, and the associations between the leukocyte, neutrophil, and lymphocyte level quartiles and the prevalence of DR in Supplementary Table 3. We also calculated the associations between the NLR level quartiles and the prevalence of diabetic complications in the same group of the participants in Supplementary Table 4. To take into account the ongoing treatment among the patients, we have reanalyzed the associations between NLR and the prevalence of CVD, DKD, and DR adjusting the further model including age, sex, education status, duration of diabetes, current smoking, BMI, HbA1c, dyslipidemia, systolic blood pressure, and the usage of antiplatelet medications in Supplementary Table 5.

## 3. Results

### 3.1. Characteristics of the Participants by the NLR Level Quartiles

Table 1 shows the general and sociodemographic characteristics of the study population. A total of 4,797 diabetic participants, with a mean age of 67 years old (SD 9, max 99, min 23), were enrolled in this study. Participants in the highest NLR level quartile, compared with those in the lowest quartile, were more likely to be men, have an older age and longer duration of diabetes, be a current smoker, and have lower education and eGFR, greater FPG, HbA1c, and urine ACR and higher prevalence of CCA plaque, CVD, DKD, and hypertension (all *P* for trend < 0.05).

### 3.2. Associations between the NLR Level and the Prevalence of CCA Plaque and CVD

The association between an elevated NLR level and an increased prevalence of CVD and CCA plaque is found in Table 2. In the unadjusted model, compared with the first quartile of the NLR level, the odds of having CCA plaque and CVD was significantly increased by 72% and 44% for participants in the highest quartile (both *P* for trend < 0.05). After adjusting for age, sex, education status, duration of diabetes, current smoking, BMI, HbA1c, dyslipidemia, and systolic blood pressure, the associations still remained.

### 3.3. Associations between the NLR Level and the Prevalence of DKD

The associations between a higher NLR level and increased Ln ACR, decreased eGFR, and greater prevalence of DKD are found in Table 3. In the total participants, compared with the lowest quartile, individuals in the highest quartile had the highest *β* for Ln ACR [0.59 (0.48, 0.70)] and the lowest *β* [-6.19 (-7.65, -4.73)] for eGFR in the unadjusted model. Furthermore, the prevalence of DKD among the participants in the highest quartile increased by 160% (OR 2.60; 95% CI 2.08, 3.24) compared with the participants in the first quartile significantly (*P* for trend < 0.001). After adjusting for age, sex, education status, duration of diabetes, current smoking, BMI, HbA1c, dyslipidemia, and systolic blood pressure, the associations of the NLR level with Ln ACR and the prevalence of DKD still remained (both *P* for trend < 0.01). After adjusting for education status, duration of diabetes, current smoking, BMI, HbA1c, dyslipidemia, and systolic blood pressure, 1SD increment of the NLR level was still significantly related to eGFR (*β* -1.85, 95% CI -2.39, -1.30).

Interestingly, in participants with normal eGFR (eGFR ≥ 90 mL/min per 1.73 m^2^), these results were replicated. The NLR level was still associated with Ln ACR and DKD positively (both *P* for trend < 0.05), indicating that the systemic inflammation indicated by the NLR level may have negative effects on nephrotic changes in the very early stage of diabetes.

### 3.4. Associations between the NLR Level and the Prevalence of DR

No association was found between the NLR level and the prevalence of DR in Table 4. In the unadjusted model, the associations of the NLR level quartiles with the prevalence of DR, NPDR, and PDR were not found. After adjusting for age, sex, education status, duration of diabetes history, current smoking, BMI, HbA1c, dyslipidemia, and systolic blood pressure, there were also no associations between the NLR level and the prevalence of DR, NPDR, and PDR (all *P* for trend > 0.05).

### 3.5. Receiver Operating Characteristics (ROC) Curve Analysis

**Figure 2:**
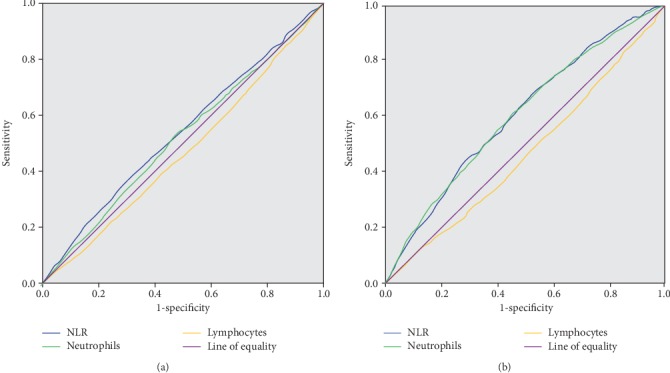
ROC curve of NLR, neutrophil, and lymphocyte levels for diagnosing CVD and DKD. (a) The ROC curve of NLR, neutrophil, and lymphocyte levels for diagnosing CVD. (b) The ROC curve of NLR, neutrophil, and lymphocyte levels for diagnosing DKD. ROC: receiver operating characteristic; NLR: neutrophil-to-lymphocyte ratio; CVD: cardiovascular and cerebrovascular diseases; DKD: diabetic kidney disease.

Figure 2(a) shows the diagnostic ability of NLR, neutrophil, and lymphocyte levels for CVD analyzed by the ROC curve. The area under ROC curve of NLR, neutrophil, and lymphocyte levels for CVD was 0.545, 0.524, and 0.463, respectively (all *P* < 0.05). The cutoff with the biggest Youden index of NLR was 2.1 with the sensitivity of 37.2% and specificity 70.5%.

The diagnostic ability of NLR, neutrophil, lymphocyte, and leukocyte levels for DKD was analyzed by the ROC curve (Figure 2(b)). The area under ROC curve of NLR, neutrophil, and lymphocyte levels for DKD was 0.607, 0.600, and 0.468, respectively (all *P* < 0.01). The cutoff with the biggest Youden index of NLR was 1.7 (sensitivity of 68.0% and specificity 47.8%).

### 3.6. Sensitivity Analyses

In Supplementary Table 1, although leukocyte and neutrophil levels were positively associated with the prevalence of CCA plaque (both *P* for trend < 0.05), no associations were found between them and the prevalence of CVD (both *P* for trend > 0.05) after adjusting for age, sex, education status, duration of diabetes history, current smoking, BMI, HbA1c, dyslipidemia, and systolic blood pressure. Lymphocytes were associated with neither the prevalence of CVD nor CCA plaque after adjusting for the same model (both *P* for trend > 0.05). In Supplementary Table 2, compared with the first quartile of leukocyte and neutrophil levels, individuals in the fourth quartile had higher prevalence of DKD, respectively [(OR 1.52; 95% CI 1.19, 1.93) and (OR 2.15; 95% CI 1.68, 2.75)] after adjusting for potential confounders (both *P* for trend < 0.05). However, compared with the first quartile of the lymphocyte level, the individuals in the fourth quartile had a lower prevalence of DKD (OR 0.61; 95% CI 0.48, 0.77) after adjusting for the same model. In Supplementary Table 3, after adjusting for potential confounders, leukocyte, neutrophil, and lymphocyte levels were all not associated with the prevalence of DR (all *P* for trend > 0.05). The different associations between the NLR level and the prevalence of CVD, DKD, and DR were also evaluated in the same population (Supplementary Table 4). A higher NLR level was still associated with an increased prevalence of CVD and DKD (both *P* for trend < 0.05), other than DR (*P* for trend > 0.05). After fully adjusting the further model including age, sex, education status, duration of diabetes, current smoking, BMI, HbA1c, dyslipidemia, systolic blood pressure, and the usage of antiplatelet medications, the associations of the NLR level with the prevalence of CVD and DKD still remained (Supplementary Table 5).

## 4. Discussion

The present study provides evidence about the associations between the NLR level and diabetic complications including CVD, DKD, and DR. The main finding was that the NLR level was positively associated with CVD and DKD, other than DR, after correction for the potential confounders. To the best of our knowledge, it is the first large-scale population study that evaluated the association between the NLR level and three chronic vascular complications in the same population simultaneously.

Accumulated evidences have implicated that chronic inflammation plays a dominant role in the development of diabetic complications [6, 28]. NLR, a readily available and inexpensive index calculated by blood routine examination, has been considered a novel inflammatory biomarker reflecting both adaptive immune response (mediated by lymphocytes) and innate immune response (mediated by neutrophils) [29, 30]. Thus, evaluating the associations between the NLR level and different diabetic complications is important.

Most of the previous studies have indicated NLR as a risk factor of CVD [31–33]. However, the results are not consistent. For example, Gijsberts et al. considered that NLR was of a limited predictive value in prediction of cardiovascular mortality [16]. Studies have suggested that presence of CCA plaque could predict CVD events [34, 35]; thus, we used it to assess early CVD risk. In our study, although the leukocyte and neutrophil levels were associated with the prevalence of CCA plaque, no association between leukocyte and the neutrophil level and the prevalence of CVD was found (Supplementary Table 1). Only the NLR level was positively associated with both CVD and CCA plaque, which suggests that the elevated NLR level may be a more proper predictor of CVD events than leukocyte and neutrophil levels. In addition, the reason for the different results regarding the associations of leukocyte and neutrophil levels between the CCA plaque and CVD may be that abnormalities of leukocytes or neutrophils have been reported in conjunction with atherosclerotic vascular diseases [36], just a predicting factor of CVD, which cannot represent the total incidence of CVD.

In the present study, we found that the NLR level was associated with the ACR level and the prevalence of DKD positively, with eGFR negatively. Similarly, our results showed, in participants with normal eGFR (eGFR ≥ 90 mL/min per 1.73 m^2^), the positive association of the NLR level with increased ACR and a higher prevalence of DKD sustained. This indicates that the change of the NLR level may start in diabetic adults with pure proteinuria. Our findings were inconsistent with the previous studies [15, 37]; both of which reported that increased NLR was significantly associated with DKD. Interestingly, we found that the neutrophil level was positively associated with the ACR level and the prevalence of CVD, whereas the lymphocyte level was associated with the ACR level and the prevalence of DKD negatively (Supplementary Table 2), which may be resulted from the harmful effects of neutrophils on endothelial damage and the antiatherosclerotic role of lymphocytes [8]. Thus, an index including both neutrophil and lymphocyte levels, like NLR, was needed vitally. We suggested that the NLR level may have better stability than independent neutrophil, lymphocyte, and leukocyte levels, since it indicates the balance between the neutrophil and lymphocyte levels, which is less affected by various pathological and physiological status.

At present, the association between the NLR level and the prevalence of DR is controversial. A hospital-based cross-sectional study indicated that NLR could be recommended as an inexpensive diagnostic biomarker for DR [38]; however, the result of the study conducted by Ciray et al. showed there was no independent association between NLR and DR [15], which our result is consistent with. The controversial results may be resulted on account of different conditions among the participants. To evaluate the different associations between the NLR level and diabetic complications, we further analyzed the associations in the same group of the participants (Supplementary Table 4). The results showed that a higher NLR level was still associated with an increased prevalence of CVD and DKD, other than DR, which suggested that the systemic inflammation indicated by the NLR level was more harmful to CVD and DKD than DR. Our result may partly explain the different incidence of CVD, DKD, and DR and may have certain enlightening effect on investigating the pathophysiological mechanisms of different diabetes complications.

There were some limitations in the present study, although it is an investigation of large sample community dwelling participants with strong quality control. First, this is a cross-sectional study; thus, causal relationships between NLR and diabetic complications cannot be confirmed. Second, our study participants were all Han Chinese, which restricted the extrapolation of current results to other ethnicities. Third, we were not able to account for all factors which might have limited the multivariate approach.

## 5. Conclusions

We found that a higher NLR level was associated with an increased prevalence of CVD and DKD, other than DR, after adjusting for the potential confounders in Chinese adult with diabetes, which suggests measuring routinely analyzed leukocyte characteristics timely may be critical for the prevention of diabetic vascular complications. Further studies are still needed to confirm present results.

## Figures and Tables

**Table 1:** Characteristics of the participants by the NLR level quartiles.

	NLR level				
Characteristic	Quartile 1 (≤1.382)	Quartile 2 (>1.382, ≤1.777)	Quartile 3 (>1.777, ≤2.319)	Quartile 4 (>2.319)	*P* for trend
*N*	1194	1211	1198	1194	/
Age (yr)	66.16 ± 8.38	67.06 ± 8.61	66.69 ± 8.40	68.73 ± 9.04	<0.001
Men (%)	38.9	43.8	48.2	53.6	<0.001
Duration of diabetes (yr)	8 (3-15)	8 (3-15)	8 (3-15)	10 (5-18)	<0.001
Current smoking (%)	14.3	18.4	19.7	19.9	<0.001
Beyond high school education (%)	53.7	52.6	52.2	48.6	0.016
BMI (kg/m^2^)	24.67 ± 3.51	25.07 ± 3.60	25.26 ± 3.58	24.84 ± 3.67	0.134
FPG (mmol/L)	7.46 ± 2.13	7.66 ± 2.40	7.90 ± 2.45	8.06 ± 2.77	<0.001
HbA1c (%)	7.40 ± 1.34	7.47 ± 1.36	7.54 ± 1.40	7.55 ± 1.41	0.005
Total cholesterol (mmol/L)	5.38 ± 1.19	5.13 ± 1.18	5.05 ± 1.19	4.90 ± 1.19	<0.001
Triglycerides (mmol/L)	1.60 (1.11-2.32)	1.53 (1.11-2.25)	1.57 (1.13-2.17)	1.46 (1.06-2.10)	0.215
HDL (mmol/L)	1.25 ± 0.30	1.21 ± 0.28	1.19 ± 0.29	1.17 ± 0.30	<0.001
LDL (mmol/L)	3.33 ± 0.85	3.17 ± 0.84	3.12 ± 0.83	3.01 ± 0.84	<0.001
Hypertension (%)	74.3	78.1	79.3	82.8	<0.001
Dyslipidemia (%)	60.1	62.4	63.8	62.6	0.169
CCA plaque (%)	27.9	34.3	34.7	39.2	<0.001
CVD (%)	34.4	34.7	37.2	41.9	<0.001
ACR (mg/g)	11 (7-21)	12 (7-27)	14 (8-34)	17 (9-44)	<0.001
eGFR (mL/min per 1.73 m^2^)	94.17 ± 14.70	92.82 ± 15.57	92.35 ± 16.51	87.30 ± 20.12	<0.001
DKD (%)	16.6	23.4	28.1	33.8	<0.001
DR (%)	15.3	17.2	18.7	16.9	0.296
NPDR (%)	14.8	17.0	18.0	16.4	0.327
PDR (%)	0.5	0.2	0.7	0.5	0.601
Leukocyte (×10 9/L)	6.02 ± 1.51	6.26 ± 1.53	6.57 ± 1.54	7.02 ± 1.88	<0.001
Lymphocytes (×10 9/L)	2.60 ± 0.76	2.21 ± 0.55	1.99 ± 0.48	1.61 ± 0.44	<0.001
Neutrophils (×10 9/L)	2.88 ± 0.76	3.48 ± 0.87	4.00 ± 0.96	4.81 ± 1.45	<0.001
NLR	1.13 ± 0.19	1.58 ± 0.12	2.02 ± 0.15	3.08 ± 0.89	<0.001

The data are summarized as the mean ± SD or median (interquartile range) for continuous variables or as a numerical proportion for categorical variables. *P* for trend was calculated by regression tests. NLR: neutrophil-to-lymphocyte ratio; BMI: body mass index; FPG: fasting plasma glucose; HbA1c: glycated hemoglobin; HDL: high-density lipoprotein; LDL: low-density lipoprotein; CCA: common carotid artery; CVD: cardiovascular and cerebrovascular diseases; ACR: albumin to creatinine ratio; eGFR: estimated glomerular infiltration rate; DKD: diabetic kidney disease; DR: diabetic retinopathy; NPDR: nonproliferative diabetic retinopathy; PDR: proliferative diabetic retinopathy.

**Table 2:** Associations between the NLR level quartiles and the prevalence of CCA plaque and CVD.

	NLR level quartiles				*P* for trend	1SD increment of NLR
	Q1 (≤1.38)	Q2 (>1.38, ≤1.78)	Q3 (>1.78, ≤2.32)	Q4 (>2.32)		
CCA plaque	Ref.	1.37 (1.15, 1.64)	1.39 (1.17, 1.66)	1.72 (1.44, 2.04)	<0.001	1.20 (1.13, 1.27)
CVD	Ref.	1.05 (0.88, 1.24)	1.15 (0.98, 1.37)	1.44 (1.22, 1.71)	<0.001	1.22 (1.14, 1.29)
CCA plaque^1^	Ref.	1.23 (1.00, 1.50)	1.24 (1.01, 1.51)	1.25 (1.02, 1.53)	0.041	1.08 (1.01, 1.16)
CVD^1^	Ref.	0.94 (0.77, 1.13)	1.10 (0.92, 1.34)	1.21 (1.00, 1.47)	0.025	1.16 (1.09, 1.24)

The participants missing vascular measurement information (*n* = 109) were excluded. Finally, 4688 participants were involved in the analyses. Data are expressed as regression coefficients (95% CI). Logistic regression analyses were used for the associations of the NLR level with the prevalence of CVD and CCA plaque with and without adjusting the model. ^1^The model was adjusted for age, sex, education status, duration of diabetes, current smoking, BMI, HbA1c, dyslipidemia, and systolic blood pressure. CVD: cardiovascular and cerebrovascular diseases; CCA: common carotid artery; BMI: body mass index; HbA1c: glycated hemoglobin; NLR: neutrophil-to-lymphocyte ratio.

**Table 3:** Associations between the NLR level quartiles and the prevalence of DKD.

	NLR level quartiles				*P* for trend	1SD increment of NLR
	Q 1 (≤1.38)	Q 2 (>1.38, ≤1.78)	Q 3 (>1.78, ≤2.32)	Q 4 (>2.32)		
In total individuals						
Ln ACR	Ref.	0.18 (0.07, 0.29)	0.36 (0.25, 0.47)	0.59 (0.48, 0.70)	<0.001	0.23 (0.19, 0.27)
eGFR	Ref.	-1.07 (-2.52, 0.38)	-1.69 (-3.15, -0.24)	-6.19 (-7.65, -4.73)	<0.001	-2.35 (-2.87, -1.84)
DKD	Ref	1.60 (1.27, 2.02)	1.98 (1.58, 2.48)	2.60 (2.08, 3.24)	<0.001	1.35 (1.26, 1.45)
Ln ACR^1^	Ref.	0.12(0.01, 0.23)	0.28(0.17, 0.39)	0.43(0.32, 0.54)	<0.001	0.18(0.14, 0.22)
eGFR^2^	Ref.	-1.24 (-2.75, 0.27)	-1.81 (-3.33, -0.29)	-5.14 (-6.66, -3.63)	<0.001	-1.85 (-2.39, -1.30)
DKD^1^	Ref.	1.54 (1.20, 1.99)	2.06 (1.61, 2.65)	2.50 (1.95, 3.19)	<0.001	1.36 (1.25, 1.47)

In individuals with normal eGFR (eGFR ≥ 90 mL/min per 1.73 m^2^)						
Ln ACR^1^	Ref.	0.11 (-0.01, 0.22)	0.27 (0.15, 0.39)	0.29 (0.17, 0.41)	<0.001	0.12 (0.07, 0.16)
DKD^1^	Ref.	1.65 (1.18, 2.30)	2.11 (1.52, 2.93)	2.19 (1.58, 3.03)	<0.001	1.20 (1.08, 1.33)

Participants missing ACR data (*n* = 233) or subjects with kidney cancer, chronic nephritis, ≥1 RBC/high-power field or ≥1 WBC/high-power field in urine sample (n = 637) were excluded. Finally, 3927 participants were involved in the analyses. Data are expressed as regression coefficients or odds ratios (95% CI). Linear regression analysis was used for the associations of the NLR level with Ln ACR and eGFR, respectively, with and without adjusting the model. Logistic regression analyses were used for the association between the NLR level and the prevalence of DKD with and without adjusting the model. ^1^The model was adjusted for age, sex, education status, duration of diabetes, current smoking, BMI, HbA1c, dyslipidemia, and systolic blood pressure. ^2^The model was adjusted for education status, duration of diabetes, current smoking, BMI, HbA1c, dyslipidemia, and systolic blood pressure. Ln ACR: logarithmically transformed albumin to creatinine ratio; eGFR: estimated glomerular infiltration rate; DKD: diabetic kidney disease; BMI: body mass index; HbA1c: glycated hemoglobin; NLR: neutrophil-to-lymphocyte ratio.

**Table 4:** Associations between the NLR level quartiles and the prevalence of DR.

	NLR level quartiles				*P* for tend	1SD increment of NLR
	Q 1 (≤1.38)	Q2 (>1.38, ≤1.77)	Q3 (>1.77, ≤2.30)	Q4 (>2.30)		
DR	Ref.	1.15 (0.88, 1.50)	1.27 (0.98, 1.65)	1.12 (0.86, 1.45)	0.314	1.01 (0.92, 1.10)
NPDR	Ref.	1.17 (0.90, 1.53)	1.26 (0.97, 1.64)	1.12 (0.86, 1.46)	0.343	1.00 (0.90, 1.12)
PDR	Ref.	0.51 (0.09, 2.77)	1.56 (0.44, 5.56)	1.02 (0.25, 4.09)	0.630	1.13 (0.70, 1.83)
DR^1^	Ref.	1.18 (0.89, 1.57)	1.36 (1.03, 1.80)	1.09 (0.82, 1.45)	0.402	1.00 (0.91, 1.11)
NPDR^1^	Ref.	1.19 (0.90, 1.58)	1.34 (1.01, 1.77)	1.06 (0.80, 1.42)	0.530	1.00 (0.90, 1.10)
PDR^1^	Ref.	0.52 (0.09, 2.87)	1.56 (0.43, 5.59)	0.94 (0.23, 3.86)	0.712	1.08 (0.69, 1. 67)

Participants missing DR information (*n* = 1531) were excluded. Finally, 3266 participants were involved in the analyses. Data are expressed as odds ratios (95% CI). Binary logistic regression was used for analyzing the association between the NLR level and the prevalence of DR with and without adjusting the model. The associations of the NLR level with NPDR and PDR were analyzed by multinomial logistic regression with and without adjusting the model. ^1^The model was adjusted for age, sex, education status, duration of diabetes, current smoking, BMI, HbA1c, dyslipidemia, and systolic blood pressure. DR: diabetic retinopathy; NPDR: nonproliferative diabetic retinopathy; PDR: proliferative diabetic retinopathy; NLR: neutrophil-to-lymphocyte ratio.

## Data Availability

The raw data supporting the conclusions of this manuscript will be made available by the authors, without undue reservation, to any qualified researcher.

## Abbreviations

T2DM: Type 2 diabetes mellitus
CVD: Cardiovascular and cerebrovascular diseases
DKD: Diabetic kidney disease
DR: Diabetic retinopathy
NLR: Neutrophil-to-lymphocyte ratio
BMI: Body mass index
FPG: Fasting plasma glucose
HbA1c: Glycated hemoglobin
HDL: High-density lipoprotein
LDL: Low-density lipoprotein
CCA: Common carotid artery
ACR: Albumin to creatinine ratio
Ln ACR: Logarithmically transformed albumin to creatinine ratio
eGFR: Estimated glomerular infiltration rate
OR: Odds ratios
CIs: Confidence intervals.

## Supplementary Materials

Supplementary Materials Supplementary Table 1: associations between the leukocyte, neutrophil, and lymphocyte level quartiles and the prevalence of CCA plaque and CVD. Supplementary Table 2: associations between the leukocyte, neutrophil, and lymphocyte level quartiles and the prevalence of DKD. Supplementary Table 3: associations between the leukocyte, neutrophil, and lymphocyte levels. Supplementary Table 4: associations between the NLR level quartiles and the prevalence of diabetic complications. Supplementary Table 5: associations between the NLR level quartiles and the prevalence of diabetic complications adjusting for the full model including the ongoing treatment.
