# Supplementary material for: Associations between the Neutrophil-to-Lymphocyte Ratio and Diabetic Complications in Adults with Diabetes: A Cross-Sectional Study
Source: J Diabetes Res. 2020 Apr 28;2020:6219545. doi: 10.1155/2020/6219545 (PMC7206875; doi:10.1155/2020/6219545)
Supplement: Supplementary Materials — Supplementary Table 1: associations between the leukocyte, neutrophil, and lymphocyte level quartiles and the prevalence of CCA plaque and CVD. Supplementary Table 2: associations between the leukocyte, neutrophil, and lymphocyte level quartiles and the prevalence of DKD. Supplementary Table 3: associations between the leukocyte, neutrophil, and lymphocyte levels. Supplementary Table 4: associations between the NLR level quartiles and the prevalence of diabetic complications. Supplementary Table 5: associations between the NLR level quartiles and the prevalence of diabetic complications adjusting for the full model including the ongoing treatment. [file 6219545.f1.docx]

|  |  | CVD | CCA plaque |
| --- | --- | --- | --- |
| Leukocyte (×10 9 /L) | Q1 (≤ 5.31) | Ref. | Ref. |
|  | Q2 (>5.31, ≤ 6.25) | 0.86 (0.71, 1.03) | 1.01 (0.83, 1.23) |
|  | Q3 (>6.25, ≤ 7.35) | 0.91 (0.75, 1.10) | 1.02 (0.84, 1.25) |
|  | Q4 (>7.35) | 1.02 (0.84, 1.23) | 1.30 (1.06, 1.59) |
|  | *P* for trend | 0.756 | 0.015 |
|  | per SD increase | 1.02 (0.95, 1.09) | 1.08 (1.01, 1.16) |
| Neutrophil (×10 9 /L) | Q1 (≤ 2.92) | Ref. | Ref. |
|  | Q2 (>2.92, ≤ 3.60) | 0.85 (0.70, 1.03) | 1.00 (0.82, 1.22) |
|  | Q3 (>3.60, ≤ 4.44) | 1.05 (0.87, 1.28) | 1.15 (0.94, 1.41) |
|  | Q4 (> 4.44) | 1.00 (0.98, 1.003) | 1.29 (1.06, 1.58) |
|  | *P* for trend | 0.056 | 0.005 |
|  | per SD increase | 1.05 (0.98, 1.12) | 1.11 (1.03, 1.19) |
| Lymphocyte (×10 9 /L) | Q1(≤ 1.65) | Ref. | Ref. |
|  | Q2 (>1.65, ≤ 2.02) | 1.03 (0.85, 1.23) | 1.05 (0.86, 1.27) |
|  | Q3 (>2.02, ≤ 2.45) | 0.88 (0.73, 1.07) | 0.98 (0.80, 1.19) |
|  | Q4 (> 2.45) | 0.86 (0.71, 1.04) | 1.03 (0.84, 1.25) |
|  | *P* for trend | 0.069 | 0.435 |
|  | per SD increase | 0.94 (0.88, 1.01) | 1.01 (0.95, 1.07) |

Supplementary table 1. Associations between the leukocyte, neutrophil and lymphocyte level quartiles and the prevalence of CCA plaque and CVD

The participants missing vascular measurement information (n=109) were excluded. Finally, 4688 participants were involved in the analyses.

Data are expressed as regression coefficients (95%CI). Logistic regression analyses were used for the associations between the leukocyte, neutrophil and lymphocyte level quartiles and the prevalence of CCA plaque and CVD.

The model was adjusted for age, sex, education, duration of diabetes, current smoking, body mass index, HbA1c, systolic blood pressure and dyslipidemia.

CVD, cardiovascular and cerebrovascular disease; CCA, common carotid artery; BMI, body mass index; HbA1c, glycated hemoglobin; NLR: neutrophil-to-lymphocyte ratio.

Supplementary table 2. Associations between the leukocyte, neutrophil and lymphocyte level quartiles and the prevalence of DKD

|  |  | All the individuals | | | eGFR ≥90 ml/min per 1.73 m^2^ |
| --- | --- | --- | --- | --- | --- |
|  |  | DKD^1^ | Ln ACR^1^ | eGFR^2^ | Ln ACR^1^ |
| Leukocyte | Q1 (≤ 5.34) | Ref. | Ref. | Ref. | Ref. |
|  | Q2 (>5.34, ≤ 6.27) | 1.06 (0.84, 1.36) | 0.02 (-0.09, 0.13) | -1.21 (-2.72, 0.31) | 0.05(-0.07, 0.17) |
|  | Q3 (>6.27, ≤ 7.35) | 1.11 (0.87, 1.42) | 0.07 (-0.04, 0.18) | -0.61 (-2.14, 0.92) | 0.05(-0.07, 0.17) |
|  | Q4 (>7.35) | 1.52 (1.19, 1.93) | 0.19 (0.07, 0.30) | -2.94 (-4.51, -1.37) | 0.15(0.02, 0.27) |
|  | *P* for trend | 0.001 | 0.001 | 0.001 | 0.034 |
|  | per SD increase | 1.19 (1.09, 1.29) | 0.09 (0.05, 0.13) | -1.07(-1.63, -0.50) | 0.06(0.01, 0.10) |
| Neutrophil | Q1 (≤ 2.93) | Ref. | Ref. | Ref. | Ref. |
|  | Q2 (>2.93, ≤ 3.61) | 1.25(0.97, 1.61) | 0.09(-0.02, 0.20) | -0.33(-1.85, 1.18) | 0.05(-0.07, 0.17) |
|  | Q3 (>3.61, ≤ 4.45) | 1.69(1.32, 2.17) | 0.24(0.13, 0.35) | -1.68(-3.21, -0.15) | 0.20(0.08, 0.32) |
|  | Q4 (>4.45) | 2.15(1.68, 2.75) | 0.38(0.27, 0.49) | -3.82(-5.37, -2.27) | 0.27(0.14, 0.39) |
|  | *P* for trend | <0.001 | <0.001 | <0.001 | <0.001 |
|  | per SD increase | 1.31(1.21, 1.42) | 0.15(0.11, 0.19) | -1.41(-1.97, -0.85) | 0.10(0.06, 0.14) |
| Lymphocyte | Q1 (≤ 1.65) | Ref. | Ref. | Ref. | Ref. |
|  | Q2 (>1.65, ≤ 2.02) | 0.86(0.69, 1.08) | -0.06(-0.17, 0.04) | 1.38 (-0.13, 2.89) | -0.12 (-0.24, 0.01) |
|  | Q3 (>2.02, ≤ 2.46) | 0.79(0.62, 0.99) | -0.15(-0.26, -0.04) | 2.87 (1.34, 4.38) | -0.19 (-0.31, -0.06) |
|  | Q4 (>2.46) | 0.61(0.48, 0.77) | -0.27(-0.38, -0.16) | 2.23 (0.69, 3.76) | -0.24 (-0.37, -0.12) |
|  | *P* for trend | <0.001 | <0.001 | 0.001 | <0.001 |
|  | per SD increase | 0.85(0.78, 0.92) | -0.09(-0.13, -0.05) | 0.87 (0.32, 1.42) | -0.07(-0.11, -0.02) |

Participants missing ACR data (n=233) or subjects with kidney cancer, chronic nephritis, ≥1 RBC/high-power filed or ≥1 WBC/high-power filed in urine sample (n=637) were excluded. Finally, 3927 participants were involved in the analyses.

Data are expressed as regression coefficients or odds ratios (95%CI). Linear regression analysis was used for the associations of the leukocyte, neutrophil and lymphocyte level quartiles with Ln ACR and eGFR, respectively. Logistic regression analyses were used for the association of the leukocyte, neutrophil and lymphocyte level quartiles with DKD.

^1^The model was adjusted for age, sex, education, duration of diabetes, current smoking, BMI, HbA1c, dyslipidemia and systolic blood pressure.

^2^The model was adjusted for education, duration of diabetes, current smoking, BMI, HbA1c, dyslipidemia and systolic blood pressure.

Ln ACR, logarithmically transformed albumin to creatinine ratio; eGFR, estimated glomerular infiltration rate; DKD, diabetic kidney disease; BMI, body mass index; HbA1c, glycated hemoglobin; NLR, neutrophil-to-lymphocyte ratio.

|  |  | DR | NPDR | PDR |
| --- | --- | --- | --- | --- |
| Leukocyte | Q1 (≤ 5.28) | Ref. | Ref. | Ref. |
|  | Q2 (>5.28, ≤ 6.23) | 1.01 (0.77, 1.33) | 1.02 (0.77, 1.35) | 0.72 (0.12, 4.36) |
|  | Q3 (>6.23, ≤ 7.36) | 1.18 (0.90, 1.56) | 1.16 (0.88, 1.53) | 2.25 (0.54, 9.32) |
|  | Q4 (>7.36) | 0.82 (0.61, 1.10) | 0.80 (0.59, 1.08) | 1.92 (0.43, 8.56) |
|  | *P* for trend | 0.397 | 0.287 | 0.213 |
|  | per SD increase | 0.94 (0.85, 1.05) | 0.92 (0.83, 1.03) | 1.54 (1.02, 2.33) |
| Neutrophil | Q1 (≤ 2.89) | Ref. | Ref. | Ref. |
|  | Q2 (>2.89, ≤ 3.58) | 1.16 (0.88, 1.53) | 1.17 (0.89, 1.56) | 0.80 (0.17, 3.74) |
|  | Q3 (>3.58, ≤ 4.45) | 1.16 (0.88, 1.54) | 1.15 (0.86, 1.53) | 1.61 (0.44, 5.87) |
|  | Q4 (>4.45) | 0.99 (0.74, 1.32) | 1.00 (0.74, 1.34) | 0.79 (0.17, 3.74) |
|  | *P* for trend | 0.926 | 0.915 | 0.925 |
|  | per SD increase | 0.96 (0.87, 1.07) | 0.95 (0.85, 1.05) | 1.44 (0.95, 2.18) |
| Lymphocyte | Q1 (≤ 1.65) | Ref. | Ref. | Ref. |
|  | Q2 (>1.65, ≤ 2.02) | 1.04 (0.79, 1.36) | 1.00 (0.76, 1.32) | 3.53 (0.70, 17.84) |
|  | Q3 (>2.02, ≤ 2.45) | 0.93 (0.70, 1.23) | 0.91 (0.69, 1.20) | 2.27 (0.41, 12.68) |
|  | Q4 (>2.45) | 0.87 (0.66, 1.16) | 0.85 (0.64, 1.13) | 2.39 (0.42, 13.68) |
|  | *P* for trend | 0.255 | 0.207 | 0.531 |
|  | per SD increase | 0.97 (0.88, 1.07) | 0.96 (0.87, 1.06) | 1.18 (0.83, 1.67) |

Supplementary table 3. Associations between the leukocyte, neutrophil and lymphocyte level quartiles and the prevalence of DR

Participants missing DR information (n=1531) were excluded. Finally, 3266 participants were involved in the analyses.

Data are expressed as odds ratios (95%CI). Binary logistic regression was used for analyzing the association between the leukocyte, neutrophil and lymphocyte level quartiles and the prevalence of DR. The associations of the leukocyte, neutrophil and lymphocyte level quartiles with NPDR and PDR were analyzed by multinomial logistic regression.

The model was adjusted for age, sex, education status, duration of diabetes, current smoking, BMI, HbA1c, dyslipidemia and systolic blood pressure.

DR, diabetic retinopathy; NPDR, nonproliferative diabetic retinopathy; PDR, proliferative diabetic retinopathy; NLR, neutrophil-to-lymphocyte ratio.

Supplementary table 4. Associations between the NLR level quartiles and the prevalence of diabetic complications

|  | NLR level quartiles | | | | *P* for tend | 1SD increment of SUA |
| --- | --- | --- | --- | --- | --- | --- |
|  | Q 1 (≤ 1.38) | Q 2 (>1.38, ≤ 1.77) | Q 3 (>1.77, ≤ 2.30) | Q 4 (> 2.30) |  |  |
| CVD | Ref. | 1.05 (0.82, 1.34) | 1.12 (0.87, 1.43) | 1.28 (1.01, 1.64) | 0.039 | 1.15 (1.06, 1.26) |
| DKD | Ref. | 1.46 (1.07, 2.01) | 2.15 (1.59, 2.92) | 2.51 (1.86, 3.40) | <0.001 | 1.37 (1.24, 1.51) |
| DR | Ref. | 1.22 (0.90, 1.65) | 1.40 (1.04, 1.89) | 1.09 (0.80, 1.49) | 0.425 | 1.00 (0.90, 1.12) |

Subjects missing DR information (n=1531), missing CVD information(n=23), missing ACR values (n=136) and subjects with kidney cancer, chronic nephritis or ≥1 WBC/high-power filed in urine sample (n=349) were excluded. Finally, 2758 subjects were included.

Data are expressed as regression coefficients or odds ratios (95% CI). Logistic regression analyses were used for the association between the NLR level quartiles and the prevalence of CVD, DKD and DR, respectively.

The model was adjusted for age, sex, education status, duration of diabetes, current smoking, current drinking, BMI, HbA1c, dyslipidemia and systolic blood pressure.

NLR, neutrophil-to-lymphocyte ratio; CVD, cardiovascular and cerebrovascular disease; DKD, diabetic kidney disease; DR, diabetic retinopathy.

Supplementary table 5. Associations between the NLR level quartiles and the prevalence of diabetic complications adjusting for the full model including the ongoing treatment

|  | NLR level quartiles | | | | *P* for tend | 1SD increment of SUA |
| --- | --- | --- | --- | --- | --- | --- |
|  | Q 1 (≤ 1.38) | Q 2 (>1.38, ≤ 1.77) | Q 3 (>1.77, ≤ 2.30) | Q 4 (> 2.30) |  |  |
| CVD | Ref. | 0.90 (0.74, 1.10) | 1.12 (0.92, 1.37) | 1.17 (0.96, 1.42) | 0.033 | 1.16 (1.08, 1.24) |
| DKD | Ref. | 1.56 (1.20, 2.03) | 2.10 (1.62, 2.73) | 2.44 (1.89, 3.15) | <0.001 | 1.35 (1.24, 1.46) |
| DR | Ref. | 1.17 (0.89, 1.56) | 1.37 (1.04, 1.80) | 1.08 (0.81, 1.44) | 0.407 | 1.00 (0.91, 1.11) |

4688, 3927 and 3266 participants were involved in the analyses for the association between NLR and CVD, NLR and DKD, NLR and DR respectively.

Data are expressed as regression coefficients or odds ratios (95% CI). Logistic regression analyses were used for the association between the NLR level quartiles and the prevalence of CVD, DKD and DR, respectively.

The model was fully adjusted for age, sex, education status, duration of diabetes, current smoking, BMI, HbA1c, dyslipidemia, systolic blood pressure, and the usage antiplatelet medications.

NLR, neutrophil-to-lymphocyte ratio; CVD, cardiovascular and cerebrovascular disease; DKD, diabetic kidney disease; DR, diabetic retinopathy.
